# Bistable Mechanisms for Space Applications

**DOI:** 10.1371/journal.pone.0168218

**Published:** 2016-12-28

**Authors:** Shannon A. Zirbel, Kyler A. Tolman, Brian P. Trease, Larry L. Howell

**Affiliations:** 1 Vehicle Systems Division, Aerospace Corporation, El Segundo, CA, United States of America; 2 Department of Mechanical, Industrial, and Manufacturing Engineering, The University of Toledo, Toledo, OH, United States of America; 3 Department of Mechanical Engineering, Brigham Young University, Provo, UT, United States of America; Semmelweis Egyetem, HUNGARY

## Abstract

Compliant bistable mechanisms are monolithic devices with two stable equilibrium positions separated by an unstable equilibrium position. They show promise in space applications as nonexplosive release mechanisms in deployment systems, thereby eliminating friction and improving the reliability and precision of those mechanical devices. This paper presents both analytical and numerical models that are used to predict bistable behavior and can be used to create bistable mechanisms in materials not previously feasible for compliant mechanisms. Materials compatible with space applications are evaluated for use as bistable mechanisms and prototypes are fabricated in three different materials. Pin-puller and cutter release mechanisms are proposed as potential space applications.

## 1 Introduction

Bistable mechanisms are proposed as a potential solution for latching or deploying space systems such as deployable solar arrays [1]. When a deployable structure is in its stowed configuration, a compliant bistable mechanism may be employed as a release device. Smaller bistable mechanisms may be embedded into the matrix of the substrate to functionally lock the structure in its deployed configuration. Creating such mechanisms using compliant mechanism theory results in devices that are easily fabricated and do not create friction or require lubrication.

Compliant mechanisms perform their function through the elastic deflection of their members. The advantages of compliant mechanisms include increased performance, reduced or eliminated assembly, no friction or wear, fewer parts, lower cost, and lower weight. These advantages make compliant mechanisms ideally suited for space or aerospace applications, where low weight and no lubrication are desirable [2].

**Fig 1:**
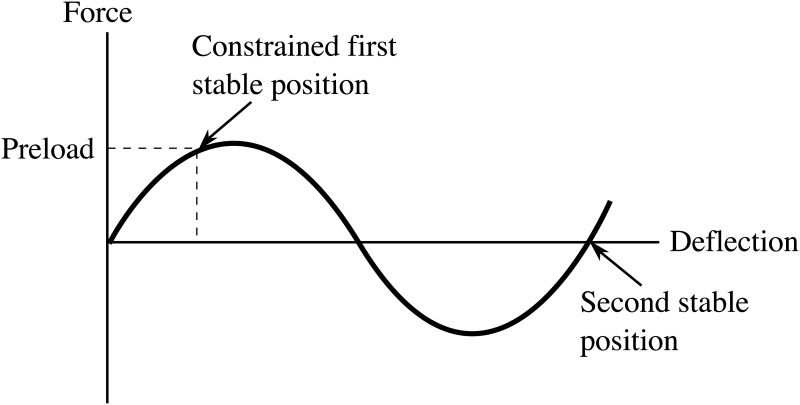
The force-deflection response for a typical bistable mechanism is shown. An optional preload stabilizes the mechanism for lower force inputs.

Compliant bistable mechanisms [3, 4] gain their bistable behavior from the energy stored in the flexible segments, which deflect to allow mechanism motion. This approach integrates desired mechanism motion and energy storage to create bistable mechanisms with dramatically reduced part count compared to traditional mechanisms incorporating rigid links, joints, and springs. As a deflection is applied to the mechanism, it rapidly transitions from one stable position to the next. The force-deflection response for a typical bistable mechanism is illustrated in Fig 1. An optional preload stabilizes the mechanism for lower force inputs.

Compliant bistable mechanisms can be used in space applications as switches, latches, or relays, thereby eliminating friction and improving the reliability and precision of those mechanical devices. Further, the bistable mechanism does not require power to be held in either of its stable positions. Such mechanisms could be integrated into deployment systems as non-explosive release mechanisms.

## 2 Background

### 2.1 Compliant Space Mechanisms

Compliant mechanisms have many advantages for space or aerospace applications and significant performance gains are possible with the introduction of compliant mechanism technology [2]. Current space-related applications of compliant mechanisms are largely limited to flexures in precision instruments such as optics. Flexures were also used in the wheels of the Mars Science Laboratory and Mars Exploration Rovers to provide suspension. Flexures have also been used to compensate for different coefficients of thermal expansion in different materials [5]. A compliant hinge providing 90 degrees of rotation was recently developed as a potential hinge for deployable booms on spacecraft [6] and compliant elements have been proposed for use in deploying booms [7].

### 2.2 Bistable Mechanisms

**Fig 2:**
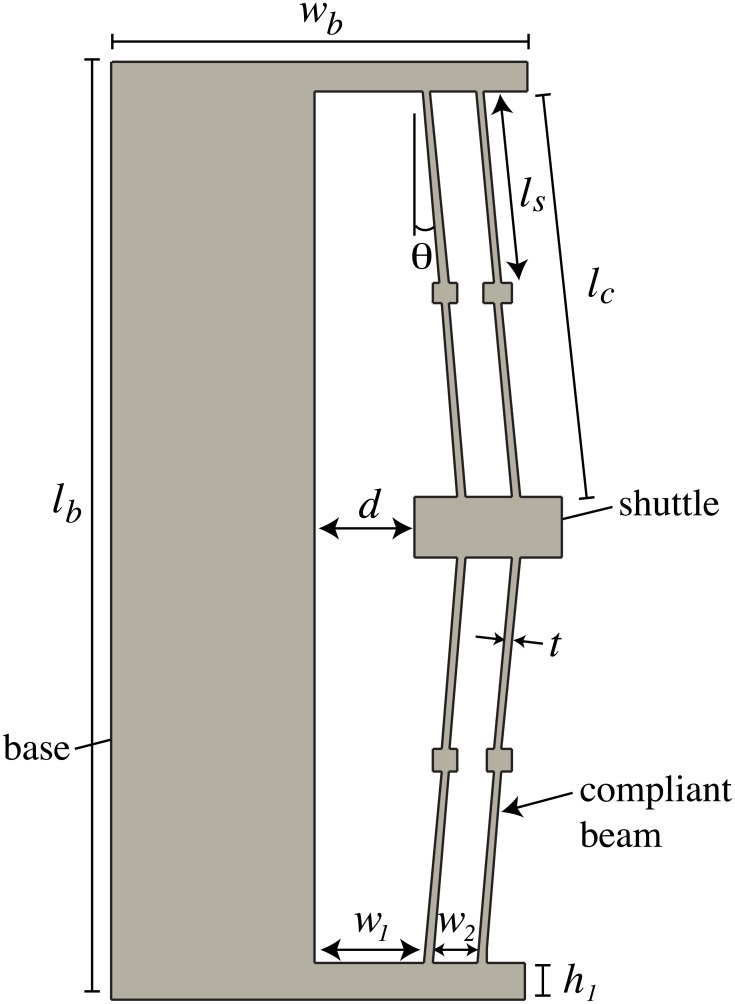
Labeled bistable mechanism.

**Fig 3:**
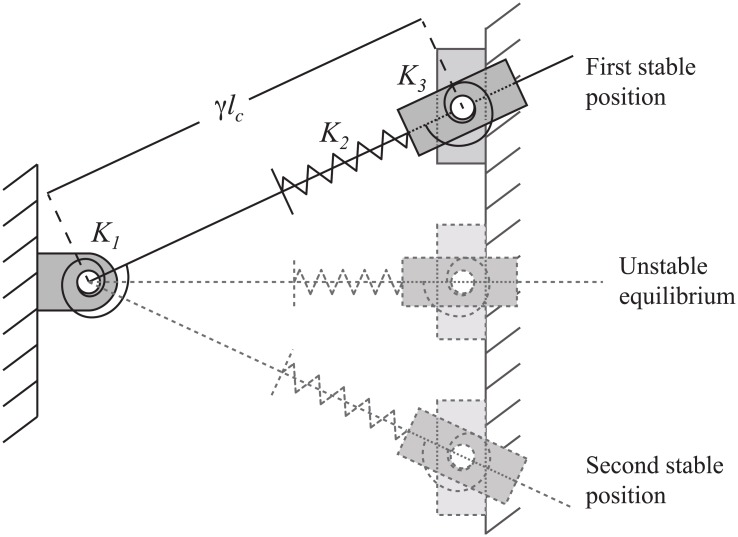
Pseudo-rigid-body model of the compliant leg. The compliance is modeled in the torsional and linear springs.

**Fig 4:**
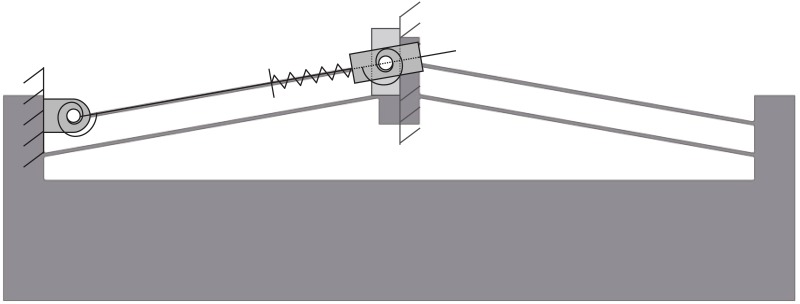
Overlay of the PRBM on the bistable mechanism.

Compliant mechanisms can achieve bistable motion without bearings or friction. They can be designed to provide precise state positions. Compliant bistable mechanisms, such as that shown in Fig 2, have potential application in space systems as switches, latches, or as an alternative to pyromechanical release devices [8–11]. Bistable mechanisms are flexible devices with two stable equilibrium positions. A pseudo-rigid-body model (PRBM) [12] is shown for a generic translating compliant bistable mechanism in Fig 3. The PRBM is overlaid on the bistable mechanism in Fig 4. The compliance in the mechanism is modeled by the inclusion of torsional and linear springs where

$$K_{1}=K_{3}=\frac{2\gamma K_{\Theta }EI}{l_{c}}$$ (1)

$$K_{2}=\frac{3EI_{b}}{w_{1}^{3}}$$ (2)

with *K*_Θ_ = 2.67617 and *γ* = 0.8517. Further, for the bistable mechanism labeled in Fig 2, *I* = *bt*^3^/12 and $I_{b}=bh_{1}^{3}/12$, where *b* is the material thickness (into the page). The PRBM is useful for initial design to find bistable configurations; then finite element analysis (FEA) is valuable to verify and refine the design. The PRBM gives reasonably accurate deflections and rougher approximations for stress.

Compliant bistable mechanisms [3, 4, 13–15] take advantage of stable minimum-energy points in their geometrically nonlinear elastic energy curves. These mechanisms are specifically engineered so the energy stored in the deflected mechanism can be quickly released when the device is actuated. This approach integrates desired mechanism motion and energy storage to create bistable mechanisms with dramatically reduced part count compared to traditional mechanisms. As a deflection is applied to the mechanism, it rapidly transitions from one stable position to the next, as illustrated in Fig 1.

Bistable mechanisms have an established history, especially in micro devices [13, 16–18]. Their application to macro devices, particularly in metals, and for space applications is a subject of current interest. The Bearing Active Preload System (BAPS) [19, 20] uses bistable mechanisms to apply a high preload during launch and a low preload during flight. The bistable mechanisms are SMA-actuated, but must be manually reset in the current design. The ability of bistable mechanisms to maintain two distinct positions without requiring external energy has shown promising applications in robotics [21–23]. Bistable mechanisms together with dielectric elastomer actuators were proposed for robotics for planetary exploration [24, 25]. They have also been proposed for use in architectural structures [26] as well as in origami-inspired structures and mechanisms [27, 28]. Tape springs have often been used in cube-sats and proposed for other space applications to enable bistability of space structures [29–31]. Bistable composites and laminates have also been developed for active shape control [32–35].

It is desirable to develop bistable mechanisms in metals because metals are more robust than polymers in many situations and can withstand the harsh environments that space imposes. Metals can withstand higher loads than polymers and are less susceptible to creep and stress relaxation. They are also thermally and electrically conductive, which can be desirable for certain applications, including actuation.

### 2.3 Release mechanisms

Release mechanisms have been developed in response to the need to anchor deployables to the spacecraft body for launch and flight, and then to be released on electrical command. Pyromechanical release devices [8–11] are common release mechanisms in aerospace applications. The have a fast response and are well understood, but are costly, one-shot devices, that apply pyro-shock loads to the spacecraft when fired.

Release mechanisms can be divided into pyromechanisms and non-explosive release mechanisms. Pyromechanisms can be further subdivided into (1) separation devices (which carry heavy loads, to be released on command), (2) cutters, and (3) pin pullers. A pyromechanism is characterized by pyro-shock, which can be from 1000-3000 *g*’s for small pyros, and up to 20,000 *g*’s for large pyros. From [8], “the load is applied so fast that the stresses at the point of application do not reach the mass of the structural element before the next increment of load is applied; i.e., the loading is being applied faster than the material can respond, and a shock wave is induced in the material.”

Non-explosive release mechanisms are characteristically slow, have lower force output, and are difficult to time. However, they do not cause shock loads like those associated with pyromechanisms. Burn-wire mechanisms, paraffin actuators, and shape-memory metal release mechanisms all have less shock, but have slower actuation times, are more complex, and can be less robust than pyromechanisms.

### 2.4 Materials selection

An integral part of designing CMs for space applications is material selection. For compliant mechanisms, we often consider the strength-to-modulus ratio of a material as a measure of its fitness for compliant applications. For space mechanisms, weight becomes critical as well. Table 1 compares the ratio of material yield strength to elastic modulus and density (*S*_*y*_/(E*ρ)) for commonly used materials in the space industry. Amorphous metals rank highest, followed by aluminum alloys 7050 and 7075, and then titanium, Elgiloy, and Inconel 718 (see Table 1). Aluminum has the lowest density of all the materials considered. Tantalum is very dense, but it is a refractory metal, highly corrosion resistant, and heat resistant. Invar, like tantalum, has a low coefficient of thermal expansion, making it ideal for optics.

**Table 1:** Strength-to-modulus and density ratios for commonly used materials in the space industry.

Material	Material Properties			Property Ratios		
	*S*_*y*_ (GPa)	*E* (GPa)	*ρ* (g/cm^3^)	*S*_*y*_/(*Eρ*) *1000	*S*_*y*_/*E* *1000	*S*_*y*_/*ρ* *1000
Vitreloy 1 (metallic glass)	1.8	95	∼5.8	3.27	18.95	310.3
Al alloy 7050	∼0.44	70.3	2.82	2.22	6.26	156
Al alloy 7075	∼0.45	71	2.8	2.26	6.34	160.7
Ti-6Al-4V	0.825	110	4.43	1.70	7.52	186.7
Elgiloy (85% HT)	2.12	189.6	8.30	1.35	11.2	255
Inconel 718 (Nickel alloy)	1.034	202.7	4.43	1.15	5.10	233
MP35N (65%)	1.62	234.8	8.43	0.82	6.90	192
Stainless 17-7TH 1050	1.034	200	7.64	0.68	5.17	135
Stainless 15-5PH H1025	0.986	196.5	7.83	0.64	5.02	126
Invar 36 (cold rolled)	0.679	148	8.05	0.57	4.59	84.3
Tantalum (UNS R05400)	0.22	185	16.6	0.07	1.19	13.2
Copper alloy (C10200-060)	0.0758	117.2	8.94	0.072	0.65	8.5
Teflon PTFE film	0.018	0.5	2.2	16.4	36	8.2
Mylar A film	0.103	3.8	1.4	19.4	27	73.5
Kapton HN film	0.07	2.8	1.42	17.6	25	49
Teflon FEP film	0.012	0.48	2.15	11.6	25	5.6
Tefzel film	0.006	1.2	1.7	3	5	3.5
Butyl rubber	0.014	3.4	0.92	4.5	4.1	15

Bulk metallic glasses (amorphous metals) are a new area of materials research. They can have high fracture toughness, although they are also characterized by a low ductility [36–40]. Metallic glasses are strong due to their lack of defined grain structure, but also have an elasticity comparable to conventional metals. The absence of microstructural defects also improves their resistance to corrosion [38]. Metallic glasses, with their exceptional yield strain, are an excellent prospect for compliant space mechanisms.

## 3 Bistable Mechanism Design

**Fig 5:**
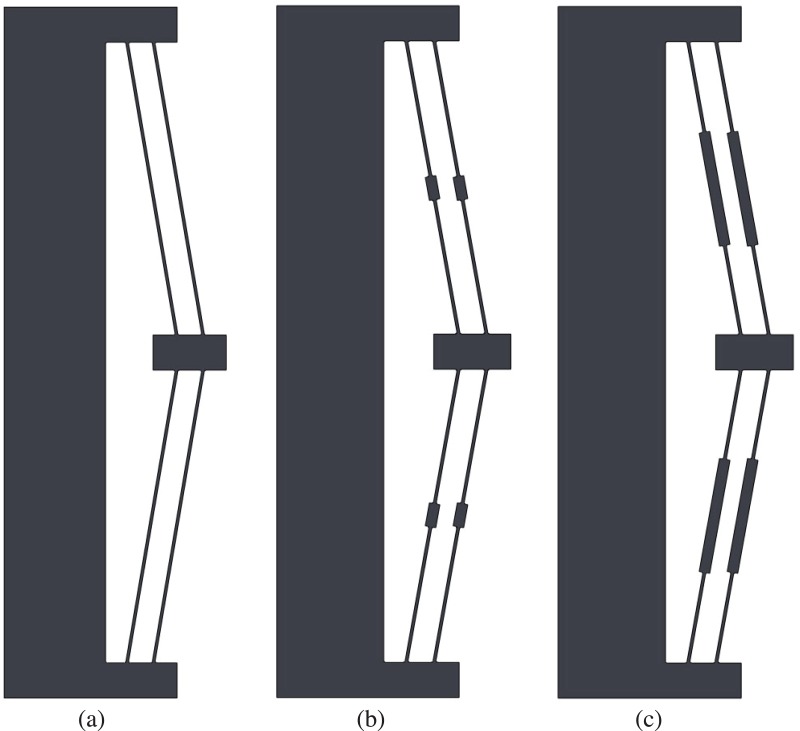
Design iterations of the compliant bistable mechanism.

Several iterations of the bistable mechanism design are illustrated in Fig 5. Fig 5a shows the basic form of the bistable device. Fig 5b and 5c show the addition of thicker midsections to the flexible beams. Such midsections are common on early bistable devices, but were included primarily because of the limitations on analytical methods; the pseudo-rigid-body model for small-length flexural pivots was originally used to model these flexures [12]. Such segments may also improve the stability of the mechanism by directing the flexible segments through a more defined motion.

**Fig 6:**
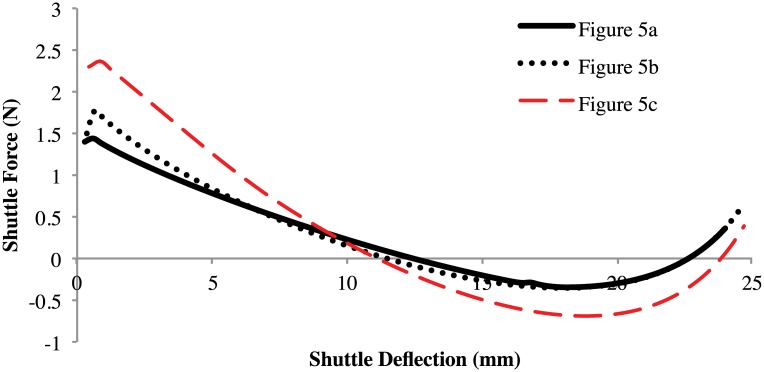
Comparing geometry of compliant segments. The thicker segments increase the force slightly.

Finite element analysis was used to determine the effect on performance of these thicker midsections. A brief analysis of the effects is shown in Fig 6 and Table 2. The thicker segments increase the bistable actuation force slightly, which helps the mechanism hold its second stable position. However, the stress is also increased with the addition of these thicker segments; the longer the thick segment, the greater the force and stress increase.

**Table 2:** Comparison of stresses for different design iterations of the compliant bistable mechanism.

		Fig 5a	Fig 5b	Fig 5c
Second stable position:	stress deflection	12.1 MPa 22.65 mm	12.2 MPa 22.64 mm	14.1 MPa 23.85 mm
Position where highest stress occurs during displacement:	stress deflection	17.6 MPa 11.66 mm	18.4 MPa 14.5 mm	29.1 MPa 5.40 mm

### 3.1 Mechanism Description

A bistable compliant mechanism was developed with properties suitable for potential application as a release mechanism in space systems. An amorphous metal, or bulk metallic glass (BMG), was selected because of its properties described earlier. As with other metals, metallic glasses are corrosion-resistant and able to withstand the harsh environment of space. It is also noteworthy that metallic glasses can be manufactured by a process similar to injection-molding for plastics [41]. This has the potential to reduce manufacturing and labor costs. The composition of the alloy selected for the design was 41.2% Zr, 13.8% Ti, 12.5% Cu, 10% Ni, and 22.5% Be (Vitreloy 1). The pertinent material properties are *S*_*y*_ = 1.8 GPa and *E* = 95 GPa. Metallic glasses have a high strength-to-modulus ratio, which is an important characteristic for compliant mechanisms because it means the material will allow a larger deflection before failure [12].

**Fig 7:**
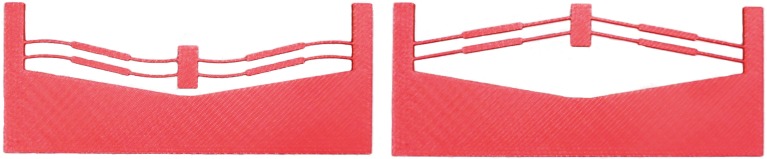
Bistable mechanism prototyped in ABS plastic, using a Dimension SST 1200ES 3D Printer, in its second stable position (left) and its fabricated position (right).

The mechanism presented in this paper was designed to evaluate the performance differences between metallic glass (specifically, Vitreloy 1) and titanium (Ti-6Al-4V). The basic design for the bistable device is illustrated in Fig 2. The material properties for Ti-6Al-4V are *S*_*y*_ = 825 MPa and *E* = 110 GPa. The part was also rapid prototyped from extruded ABS as an early demonstrator of the model (see Fig 7). The relevant material properties used for the ABS design and analysis are *S*_*y*_ = 36 MPa and *E* = 2 GPa. The design parameters are listed in Table 3.

**Table 3:** Design parameters for the bistable mechanism prototypes.

	Extruded ABS (Fig 7)	Metallic Glass (Same Safety Factor)	Titanium (Same Safety Factor)	Metallic Glass/Titanium (Same Geometry)
*d*	27 mm	10 mm	19 mm	11.5 mm
*l*_*s*_	25 mm	13 mm	36 mm	25 mm
*l*_*c*_	80.5 mm	30 mm	76 mm	55.25 mm
*l*_*b*_	192.4 mm	74 mm	166 mm	129.6 mm
*t*	0.8 mm	0.5 mm	0.88 mm	0.88 mm
*θ*	80 deg	82 deg	82 deg	82 deg
*w*_*b*_	78.5 mm	30 mm	34 mm	30 mm
*w*_1_	5 mm	6.5 mm	6.5 mm	5.6 mm
*w*_2_	6 mm	6 mm	6 mm	4.4 mm
*h*_1_	10 mm	5 mm	5 mm	8 mm

The bistability of the device is irrespective of the thickness of the material, but the actuation force is increased with increasing thickness (i.e., increasing compliant beam width). All designs were made for 3 mm thick sections. The shuttle should snap between two stable positions, with the second position bringing the shuttle just into contact with the base. Fig 2 defines the pertinent design parameters that can be changed or optimized to give a feasible design.

**Fig 8:**
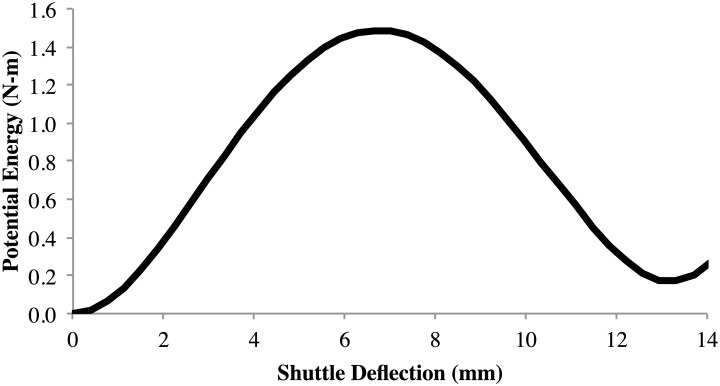
Predicted energy curve of the metallic glass bistable mechanism from the PRBM.

**Fig 9:**
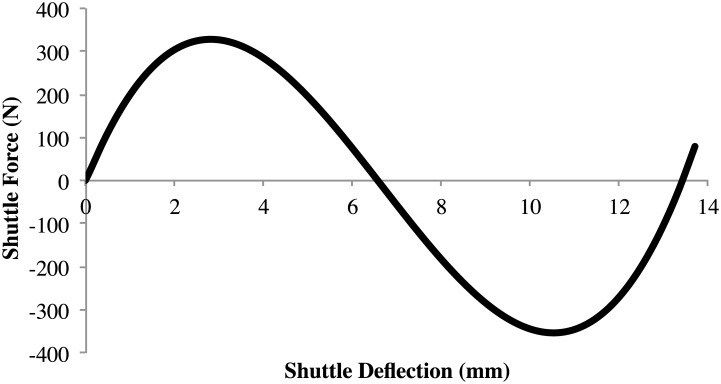
Predicted force-displacement curve of the metallic glass bistable mechanism from the PRBM.

The compliant deflection was modeled using the pseudo-rigid-body model (PRBM), as shown in Fig 3. Figs 8 and 9 show the predicted energy and force curves for the three designs, as determined by the PRBM. These models were then analyzed using finite element analysis and prototypes were fabricated and tested.

**Fig 10:**
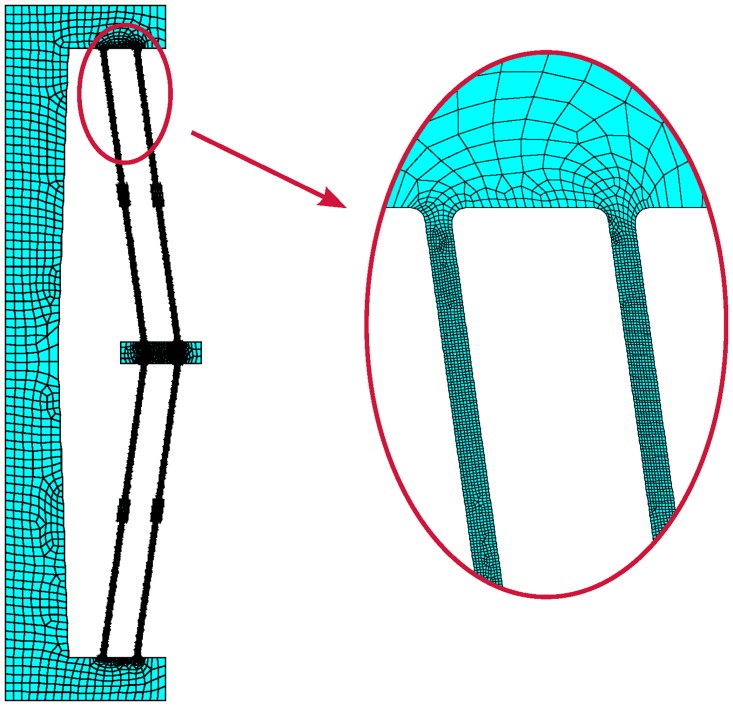
An example of the finite element mesh for the bistable mechanism.

The finite element model for the bistable mechanism is a membrane model using ANSYS PLANE182 elements with mid-side nodes. The mesh, shown in Fig 10, was created by specifying the number of divisions along each of the lines in the model. The compliant segments required a finer mesh because they will undergo large, nonlinear deflections. The outer geometry goes through less displacement and can therefore have a coarser mesh. The finite element analysis is displacement-controlled, where the total displacement was applied over 60-70 load steps, depending on which material is being modeled.

## 4 Results

Several bistable compliant mechanisms were manufactured from titanium (Ti-6Al-4V) and metallic glass (Vitreloy 1) by wire-EDM [41]. The performance of the two materials was compared through two controlled designs. The main design parameters are listed in Table 3. The two mechanisms were manufactured to maintain the same safety factor, or same ratio of the yield strength to the maximum stress. This resulted in a titanium device that was more than twice as long as that of metallic glass mechanism due to the large difference in material properties. The two mechanisms were also manufactured with identical geometries. The safety factor for the metallic glass mechanism is over two, while the safety factor for the titanium mechanism is equal to one. With such a low factor of safety, it is likely that the titanium mechanism experienced local yielding.

To compare performance, the identically sized titanium and metallic glass flexures were tested in a load frame to determine the force-displacement behavior. The results of the test are described in [41], where it can be seen that the mechanisms exhibit bistability with a clear intermediate instability point. The two materials exhibit roughly the same response because they have comparable stiffnesses, but the strength of the metallic glass is twice the strength of the titanium, thereby doubling its factor of safety.

**Fig 11:**
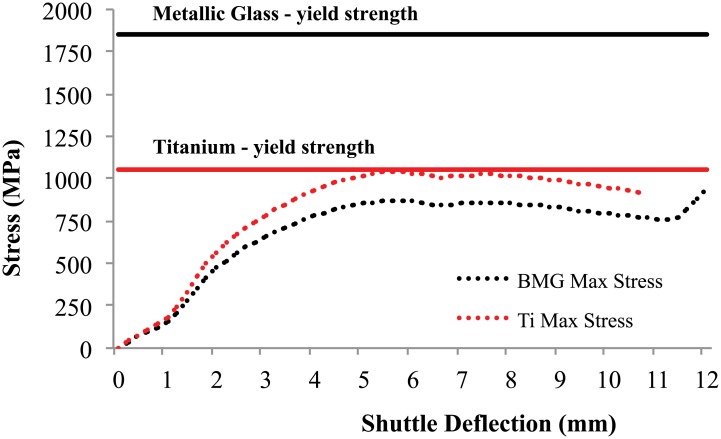
The maximum stress in the identically sized mechanisms as predicted by FEA.

Finite element analysis was used to verify and refine the design and to determine the maximum stress in the compliant members. The ANSYS finite element model consists of 10,000-20,000 PLANE182 elements, varying with the size of the mechanism. The mesh was refined along the compliant flexures and the motions were examined over 60-70 steps. The FEA results predicted a bistable response, with force and displacement predictions accurate within 80% [41]. The maximum stress in the finite element model was measured and is plotted in Fig 11. The yield strengths for metallic glass and titanium are indicated in the plot. This illustrates the difference in safety factors for the two materials with identical geometries. There is a difference between the FEA results and experimental results for the second stable equilibrium positions. The FEA assumes a rigid-fixed condition while the hardware necessarily has some elasticity even for the relatively rigid components and connections. This difference is seen most in areas of higher deformations, such as the unstable equilibrium position and the second equilibrium position.

**Fig 12:**
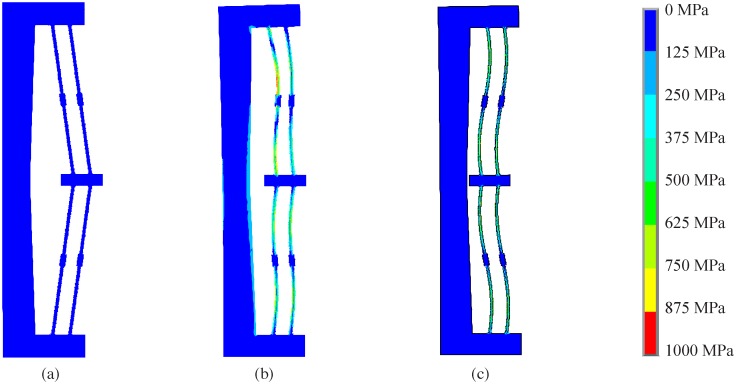
(a) Initial position of the finite element model. (b) Position where highest stress occurs during deflection. (c) Second stable position of the model.

Fig 12 shows an example of the different stress states in the finite element model during the simulated displacement. The highest stress state (Fig 12b) occurs partway through the deflection, when the compliant beams are under a high compressive load. The maximum stress state occurs in the buckled flexures; its location varies along the length of the flexure depending on how the beams buckle.

There is a complex interplay between geometry and material properties that affect bistability. Table 4 lists the key parameters for bistability, which are all interdependent. The parameters *θ*, *l*_*c*_, *t*, *h*_1_, and *w*_1_ (as defined in Fig 2) all affect bistability and stress. Table 4 summarizes the effect of these parameters on bistability and stress. We defined an improvement in bistability as as increase in the force required to reset the mechanism from its second stable position to its first (as fabricated) position. As can be seen from the table, the dimensions *l*_*c*_ and *t* will be driven by the constraints of the manufacturing process and design space; optimizing the design will drive *t* to its minimum thickness and *l*_*c*_ to its maximum allowable length.

**Table 4:** Summary of how changes in the design parameters affect bistability and stress.

To improve bistability:	To reduce stress:
Increase *θ*	Decrease *θ*
Increase *l*_*c*_	Increase *l*_*c*_
Decrease* *t*	Decrease *t*
Increase* *h*_1_	Decrease *h*_1_
Decrease *w*_1_	Increase *w*_1_

*Approaches an asymptote

## 5 Proposed Applications

Compliant bistable release mechanisms can be used as non-explosive release mechanisms at a fraction of the cost and weight of traditional release mechanisms. Compliant bistable release mechanisms would eliminate the challenges of having explosive charges on the spacecraft. They can be compact compared to other alternatives, thereby reducing weight. They will enable systems to be testable and resettable. Other advantages of compliant bistable mechanisms are that they can accommodate integrated thermal actuation to change state and they only require power to change states, not to maintain state (this means much lower power requirements than many alternatives).

Bistable mechanisms share many of the same desired characteristics of explosively actuated mechanisms in that they respond rapidly upon triggering, are capable of high force output, and can be designed so that they only require minimal amounts of activation energy to release large amounts of stored energy. A major advantage of bistable mechanisms is that the actuation is easily reversed for repeated testing and the tested hardware can be flown, which is not possible to do with pyromechanisms.

**Fig 13:**
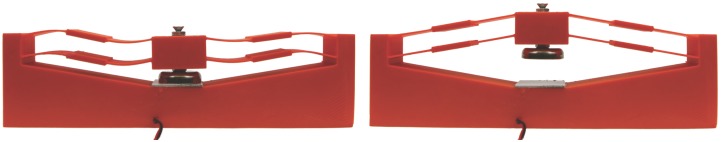
A bistable non-explosive release mechanism demonstrator in its second stable position (left) and its fabricated position (right).

Actuation of bistable mechanisms differs from that of current explosive and non-explosive release mechanisms. While it is possible that a similar method could be used to actuate bistable mechanisms, such as the use of shape-memory materials [42, 43] or the heating of the bistable flexures to trigger them into their second position, we also investigated more rapid actuation methods. Because a small input displacement can actuate a bistable mechanism from its second stable position to its fabricated position, this is one of the simplest methods of activation. A prototype of a magnetically actuated mechanism, shown in Fig 13, uses a 3A-12V electromagnetic linear actuator to demonstrate this type of actuation. In the prototype, a push-pull electromagnet was used; however, a device such as a solenoid actuator would be more ideal for the final design due to its greater efficiency. The prototype was 3D-printed out of PLA filament. The electromagnet provides a force of 8.9 N (2 lbf) at a gap of 1.6 mm (0.063 in). The prototype was designed such that the electromagnet would provide just enough force to trip the mechanism into its fabricated position from its second stable position.

The prototype in Fig 13 demonstrated the effectiveness of electromagnetic actuation of bistable mechanisms. The benefits of such a system include the fact that much less input force from the linear actuator is required to activate the system than is output by the bistable mechanism; when the mechanism is in its second stable position (Fig 13 (left)), an input force of 8.9 N actuates the mechanism and outputs 40 N. This allows a high magnitude force to be preloaded into the deployment mechanism before launch, and requires only a small electrical pulse to release the stored energy almost instantaneously.

Two basic release mechanisms, a pin-puller and a cutter, were chosen to evaluate the possibilities of using the developed bistable mechanism as an alternative to pyromechanical release mechanisms. Due to the nature of the high output force requirements, the frame of the mechanism was closed and additional compliant legs were added to the design to increase the transition force. The prototypes were 3D-printed in PLA.

**Fig 14:**
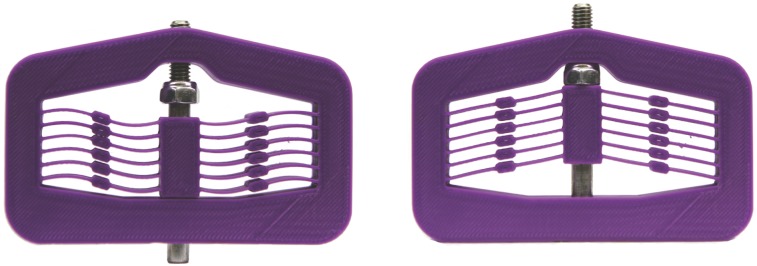
Prototype of a pin-puller non-explosive release mechanism in its second stable position (left) and its fabricated position (right).

A bistable version of a pin-puller release mechanism is shown in Fig 14. The puller is designed so the bistable mechanism makes contact with the pin only after the legs have moved well past the unstable position. The contact position is such that the bistable mechanism engages with the pin while it is in the high-force region of its displacement. In its movement from the unstable position to the pin-contact point, the bistable mechanism gains momentum which aids in breaking the pin free from the static friction caused by the loading on it. The bistable mechanism, then in its high force region, would then pull the pin free.

**Fig 15:**
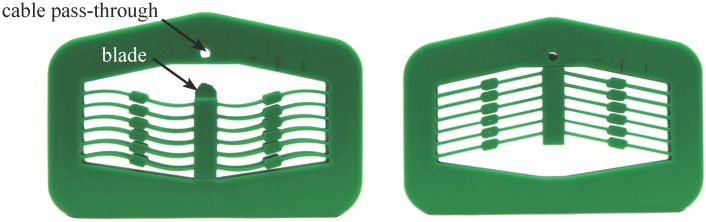
Prototype of a cutter non-explosive release mechanism in its second stable position (left) and its fabricated position (right).

The cutting mechanism demonstrator shown in Fig 15 was designed using the same bistable configuration that was developed for the puller. The additional compliant legs and the increased width of the mechanism provide a higher force for cutting wires and cables. The mechanism is designed such that the cutter engages with the wire or cable while in the high-force region of the shuttle deflection. The momentum gained in the travel from the unstable position to the engagement position aids in the initial cutting. As the blade cuts through the material, it meets the mandrel in a preloaded position that maintains high force between the blade and mandrel, thus ensuring a complete cut.

**Fig 16:**
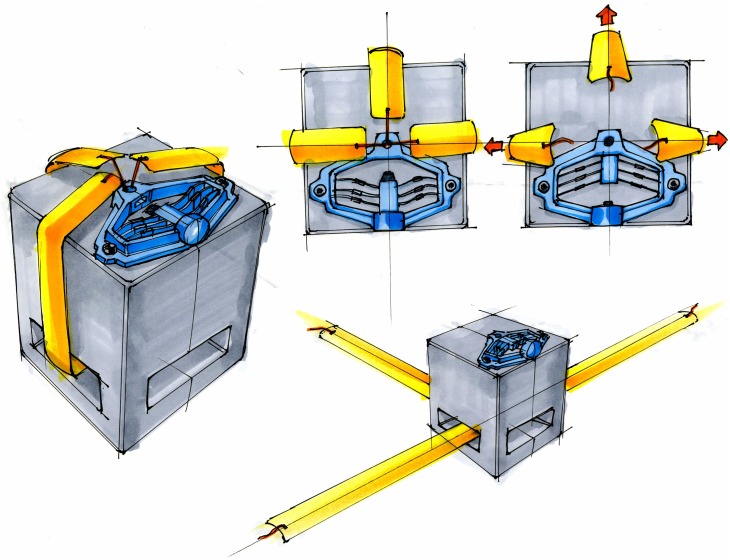
Conceptual illustration of the cable cutting mechanism being employed as an antennae release mechanism on a cube-sat.

The 3D-printed prototype release mechanisms demonstrate potential applications for bistable mechanisms. To be used in space, the bistable release mechanisms would be manufactured in bulk metallic glass or titanium. Finite element analysis reveals that if the same design used in the pin-puller and cable cutter prototypes was fabricated in BMG rather than plastic, forces of over 400 N (90 lbf) could be achieved, ensuring that these mechanisms can be tailored to meet a variety of load requirements. These mechanisms could be easily incorporated into current space vehicles and satellites without requiring major modifications. An artist’s rendition of a cable cutter for deploying antennae on a cube-sat is shown in Fig 16.

Future work would include determining the robustness of the mechanisms during launch. Stiffness of the device can be tuned so that natural frequencies do not coincide with the launch vibration spectrum. The mechanism should also be oriented perpendicular to the application of undesired loads or vibrations to reduce the chance of misfire.

## 6 Conclusion

Compliant bistable mechanisms are proposed for space applications as switches, latches, or relays, thereby eliminating friction, improving the reliability and precision, and enabling testing of these mechanisms. Such mechanisms could also be integrated into deployment systems as non-explosive release mechanisms.

Analytical and numerical methods have been presented to analyze monolithic bistable mechanisms. The models were exercised to successfully develop monolithic metal bistable mechanisms. This achievement is significant because the high stiffness of metals makes their use in monolithic bistable mechanisms challenging. This work has led to their use in space applications because of increased reliability, less variation in force response, low susceptibility to stress relaxation while in stowed configuration, and less susceptibility to temperature variations and out-gassing when compared to materials more traditionally used in monolithic bistable mechanisms.

Flight applications for compliant bistable release mechanisms include deployable structures, camera covers, launch locks, force sensors, maximum load indicators, and shutter mechanisms. The technology developed to create these devices can be extended to other areas of space-related research, including gradient alloys, amorphous metals, and flexible electronics. The technology can also be extended to other industries. The relatively simple design has the potential to reduce part count and improve the reliability of mechanical systems. In mechanisms that undergo similar motions, compliant bistable mechanisms should be considered as an alternative to current methods.

## References

[1] Zirbel S A, Lang R J, et al Accommodating thickness in origami-based deployable arrays. J Mech Des. 2013;135(11). 10.1115/1.4025372

[2] Fowler RM, Howell LL, Magleby SP. Compliant Space Mechanisms: A New Frontier for Compliant Mechanisms. Mechanical Sciences, vol 2, pp 205-215. 2011;2:205–215.

[3] Lusk CP, Howell LL. Spherical Bistable Micromechanism. J Mech Des. 2008;130(4):045001 10.1115/1.2885079

[4] Chen G, Aten QT, Zirbel S, Jensen BD, Howell LL. A Tristable Mechanism Configuration Employing Orthogonal Compliant Mechanisms. J Mechanisms Robotics. 2010;2(1):014501 10.1115/1.4000529

[5] Pollard LW. A flexure mount for optics in dynamic and thermal environments. Proceedings of the 6th International Congress on Applications of Lasers and Electro-optics. 1987;563:109.

[6] Fowler RM, Maselli A, Pluimers P, Magleby SP, Howell LL. Flex-16: A large-displacement monolithic compliant rotational hinge. Mechanism and Machine Theory. 2014;82:203–217. 10.1016/j.mechmachtheory.2014.08.008.

[7] Mallikarachchi H, Pellegrino S. Design of Ultrathin Composite Self-Deployable Booms. Journal of Spacecraft and Rockets. 2014; p. 1–11.

[8] Conley P, editor. Space Vehicle Mechanisms: Elements of Successful Design. John Wiley and Sons, Inc; 1998.

[9] Purdy WE. Advanced Release Technologies Program. Proceedings of the 28th Aerospace Mechanisms Symposium. 1994;.

[10] Tibbitts S. High Output Paraffin Actuators: Utilization in Aerospace Mechanisms. Proceedings of the 22nd Aerospace Mechanisms Symposium. 1988;.

[11] Robinson A, Courtney C, Moran T. Non-explosive Actuation for the ORBCOMM. Proceedings of the 29th Aerospace Mechanisms Symposium. 1995;.

[12] Howell LL. Compliant Mechanisms. John Wiley and Sons; 2001.

[13] Jensen BD, Howell LL, Salmon LG. Design of Two-Link, In-Plane, Bistable Compliant Micro-Mechanisms. J Mech Des. 1999;121(3):416–423. 10.1115/1.2829477

[14] Chen G, Ma F. Kinetostatic modeling of fully compliant bistable mechanisms using Timoshenko beam constraint model. Journal of Mechanical Design. 2015;137(2):022301 10.1115/1.4029024

[15] Chen G, Gou Y, Zhang A. Synthesis of compliant multistable mechanisms through use of a single bistable mechanism. Journal of Mechanical Design. 2011;133(8):081007 10.1115/1.4004543

[16] Wilcox DL, Howell LL. Fully compliant tensural bistable micromechanisms (FTBM). J Microelectromech Sys. 2005;14(6):1223–1235. 10.1109/JMEMS.2005.859089

[17] Qiu J, Lang JH, Slocum AH. A curved-beam bistable mechanism. J Microelectromech Sys. 2004;13(2):137–146. 10.1109/JMEMS.2004.825308

[18] Huang HW, Yang YJ. A MEMS Bistable Device With Push-On–Push-Off Capability. J Microelectromech Sys. 2013;22(1):7–9. 10.1109/JMEMS.2012.2228165

[19] Lewis SD, Munro G, Humphries ME, Szekely G. Development of an adjustable bearing preload enabled-optical terminal. Proceedings of the 13th European Space Mechanisms and Tribology Symposium. 2009;.

[20] Lewis S, Humphries M. Development, Pre-qualification and Application of an Active Bearing Preload System. Proceedings of the 38th Aerospace Mechanisms Symposium. 2006;.

[21] Kim SW, Koh JS, Lee JG, Ryu J, Cho M, Cho KJ. Flytrap-inspired robot using structurally integrated actuation based on bistability and a developable surface. Bioinspiration & biomimetics. 2014;9(3):036004 10.1088/1748-3182/9/3/036004 24615620

[22] Zhang Z, Chen D, Wu H, Bao Y, Chai G. Non-contact magnetic driving bioinspired Venus flytrap robot based on bistable anti-symmetric CFRP structure. Composite Structures. 2016;135:17–22. 10.1016/j.compstruct.2015.09.015

[23] Follador M, Conn A, Mazzolai B, Rossiter J. Active-elastic bistable minimum energy structures. Applied Physics Letters. 2014;105(14):141903 10.1063/1.4898142

[24] Wingert A, Lichter MD, Dubowsky S. On the design of large degree-of-freedom digital mechatronic devices based on bistable dielectric elastomer actuators. IEEE/ASME Transactions on Mechatronics. 2006;11(4):448–456. 10.1109/TMECH.2006.878542

[25] Lichter MD, Sujan VA, Dubowsky S. Experimental demonstrations of a new design paradigm in space robotics. Experimental Robotics VII Book Series: Lecture Notes in Control and Information Sciences. 2001;271:219–228. 10.1007/3-540-45118-8_23

[26] Ohsaki M, Tsuda S, Watanabe H. Optimization of Retractable Structures Utilizing Bistable Compliant Mechanism. Engineering Structures. 2013;56:910–918. 10.1016/j.engstruct.2013.06.019

[27] Silverberg JL, Na JH, Evans AA, Liu B, Hull TC, Santangelo CD, et al Origami structures with a critical transition to bistability arising from hidden degrees of freedom. Nature materials. 2015;14(4):389–393. 10.1038/nmat4232 25751075

[28] Safsten C, Fillmore T, Logan A, Halverson D, Howell L. Analyzing the Stability Properties of Kaleidocycles. Journal of Applied Mechanics. 2016;83(5):051001 10.1115/1.4032572

[29] Santer M, Pellegrino S. Compliant multistable structural elements. International Journal of Solids and Structures. 2008;45(24):6190–6204. 10.1016/j.ijsolstr.2008.07.014.

[30] Pirrera A, Avitabile D, Weaver PM. On the thermally induced bistability of composite cylindrical shells for morphing structures. International Journal of Solids and Structures. 2012;49(5):685–700. 10.1016/j.ijsolstr.2011.11.011.

[31] Schioler T, Pellegrino S. Space frames with multiple stable configurations. AIAA Journal. 2007;45(7):1740–1747. 10.2514/1.16825

[32] Kim HA, Betts DN, Salo AIT, Bowen CR. Shape memory alloy-piezoelectric active structures for reversible actuation of bistable composites. AIAA Journal. 2010;48(6):1265–1268. 10.2514/1.J050100

[33] Betts DN, Kim HA, Bowen CR. Optimization of stiffness characteristics for the design of bistable composite laminates. AIAA journal. 2012;50(10):2211–2218. 10.2514/1.J051535

[34] Daynes S, Nall S, Weaver P, Potter K, Margaris P, Mellor P. Bistable composite flap for an airfoil. Journal of Aircraft. 2010;47(1):334–338. 10.2514/1.45389

[35] Emam SA, Inman DJ. A Review on Bistable Composite Laminates for Morphing and Energy Harvesting. Applied Mechanics Reviews. 2015;67(6):060803 10.1115/1.4032037

[36] Demetriou MD, Launey ME, Garrett G, Schramm JP, Hofmann DC, Johnson WL, et al A damage-tolerant glass. Nature Materials. 2011;10:123–128. 10.1038/nmat2930 21217693

[37] Launey ME, Hofmann DC, Johnson WL, Ritchie RO. Solution to the problem of the poor cyclic fatigue resistance of bulk metallic glasses. Proceedings of the National Academy of Sciences. 2009;106(13):4986–4991. 10.1073/pnas.0900740106 19289820 PMC2663983

[38] Demetriou MD, Wiest A, Hofmann DC, Johnson WL, Han B, Wolfson N, et al Amorphous Metals for Hard-tissue Prosthesis. Journal of the Minerals, Metals, and Materials Society. 2010;62(2):83–91. 10.1007/s11837-010-0038-2

[39] Hofmann DC, Suh JY, Wiest A, Duan G, Lind ML, Demetriou MD, et al Designing metallic glass matrix composites with high toughness and tensile ductility. Nature. 2008;451:1085–1089. 10.1038/nature06598 18305540

[40] Hofmann DC. Shape Memory Bulk Metallic Glass Composites. Science. 2010;329:1294–1295. 10.1126/science.1193522 20829474

[41] Homer ER, Harris MB, Zirbel SA, Kolodziejska JA, Kozachkov H, Trease BP, et al New Methods for Developing and Manufacturing Compliant Mechanisms Utilizing Bulk Metallic Glass. Advanced Engineering Materials. 2014; p. 10.1002/adem.201300566

[42] Follador M, Cianchetti M, Mazzolai B. Design of a compact bistable mechanism based on dielectric elastomer actuators. Meccanica. 2015;50(11):2741–2749. 10.1007/s11012-015-0212-2

[43] Zhou M, Zhang Q, Wang J. Feedforward-feedback hybrid control for magnetic shape memory alloy actuators based on the Krasnosel’skii-Pokrovskii model. PloS one. 2014;9(5):e97086 10.1371/journal.pone.0097086 24828010 PMC4020807

